# Association between pre-treatment malnutrition and chemotherapy toxicity in patients with advanced or metastatic gastroenteric tumors receiving systemic therapy

**DOI:** 10.3389/fnut.2026.1780440

**Published:** 2026-06-11

**Authors:** Yadong Dong, XiaoQiang Yan, Qianqian Ma, Dongqing Liu, Lin Wang, Mingyuan Li, Jie Zhao

**Affiliations:** 1 National Engineering Laboratory for Internet Medical Systems and Applications, The First Affiliated Hospital of Zhengzhou University, Zhengzhou, China; 2 School of Computer and Artificial Intelligence, Zhengzhou University, Zhengzhou, China; 3 Institute of Intelligent Medicine, Henan Academy of Innovations in Medical Science, Zhengzhou, China

**Keywords:** hematological toxicity, malnourishment, nutritional risk index, prognostic nutritional index, stomach cancer

## Abstract

**Background:**

Malnutrition is prevalent in individuals with gastroenteric malignancies and may negatively influence chemotherapy tolerance. This investigation evaluated the relationships between pre-treatment nutritional status and chemotherapy-derived toxicity utilizing anthropometric, biochemical, and composite nutritional indices.

**Methods:**

A retrospective analysis was conducted involving 720 individuals with histologically verified advanced or metastatic gastroenteric malignancies, including 360 gastric cancer cases and 360 colorectal cancer cases, who were given systemic cytotoxic chemotherapy between 2017 and 2023. Baseline nutritional status was examined via body mass index (BMI), serum albumin, Prognostic Nutritional Index (PNI), and Nutritional Risk Index (NRI). Chemotherapy-associated toxicities were graded based on the CTCAE criteria. Multivariable logistic regression models were built individually by cancer type and toxicity category.

**Results:**

Hematological toxicity was reported in 49.7% of gastric cancer and 63.1% of colorectal cancer subjects, while non-hematological toxicity occurred in 38.1 and 56.1%, respectively. In gastric cancer, underweight status significantly elevated the possibility of hematological toxicity (OR 1.97, 95% CI 1.06–3.64; *p* = 0.03), as did hypoalbuminemia (OR 1.71, 95% CI 1.11–2.62; *p* = 0.01) and PNI ≤ 45 (OR 1.70, 95% CI 1.11–2.61; *p* = 0.01). Age was independently linked with non-hematological damage (OR 1.03 per year, 95% CI 1.01–1.06; *p* = 0.001). In colorectal cancer, age individually indicated both hematological (OR 1.03 per year; *p* = 0.001) and non-hematological harm (OR 1.04 per year; *p* = 0.001). Lower BMI (OR 2.00, 95% CI 1.02–3.91; *p* = 0.04), hypoalbuminemia (OR 1.85, 95% CI 1.12–3.04; *p* = 0.01), PNI ≤ 45 (OR 1.90, 95% CI 1.21–2.97; *p* = 0.005), and NRI ≤ 83 (OR 2.50, 95% CI 1.29–4.85; *p* = 0.007) individually determined hematological injury, while hypoalbuminemia (OR 1.90; *p* = 0.01) and NRI ≤ 83 (OR 2.35; *p* = 0.01) were also linked with non-hematological damage.

**Conclusion:**

Pre-treatment nutritional inadequacy was connected with chemotherapy toxicity, which supports periodic nutritional screening prior to medication.

## 1 Introduction

Malnutrition is a prevalent and clinically serious situation amongst cancer patients with an exceptionally high incidence in individuals that have been diagnosed with gastroenteric malignancies. Gastrointestinal tract tumors expose the patient to nutritional impairment, which could occur because of decreased oral intake, obstruction caused by the tumor, malabsorption, malmetabolic state, and systemic inflammation. According to previous studies and systematic reviews, the prevalence of malnutrition is 30 to over 70 percent in gastric, colorectal, pancreatic, and other gastrointestinal cancers (1, 2). Treatment of gastroenteric tumors rests on the management with chemotherapy which is often accompanied with severe toxicities that impair treatment tolerance and treatment outcomes. Adverse events, such as myelosuppression, gastrointestinal toxicity, fatigue, mucositis, infections, and discontinuation of treatment induced by chemotherapy are still central clinical problems. There is also an increasing body of evidence showing that malnutrition in pre-treatment condition is a significant risk factor of severe chemotherapy toxicity, early reduction of dosing, delay of treatment, hospitalization, and early mortality (3, 4).

A number of observational and prospective studies have shown a close relationship between adverse events related to chemotherapy, and poor nutritional indices. The initial report of an association between malnutrition-related indices with chemotherapy-related toxicity was made previously, confirming that the body mass index (BMI), prognostic nutritional index (PNI), and weight loss were significant independently (5). These findings have been extended in gastroenteric malignancies through subsequent research. Both nutritional and sarcopenic parameters were found to be relevant as low PNI, nutrition risk index (NRI), and skeletal muscle index were significant predictors of early myelosuppression in stage IV gastric cancer (6). Likewise, studies indicated that impaired nutritional health had a negative impact on chemotherapy compliance, response level, and survival of metastatic colorectal cancer (7, 8).

In addition to PNI and NRI, a number of well-known nutritional assessment instruments are frequently used in oncology, such as the Mini Nutritional Assessment (MNA), the Global Leadership Initiative on Malnutrition (GLIM) criteria, the Patient-Generated Subjective Global Assessment (PG-SGA), and the Nutritional Risk Screening 2002 (NRS-2002). These methods cover a broader range of functional, phenotypic, and clinical aspects and have shown prognostic utility in cancer populations. Specifically, hospital-based studies employing structured evaluation approaches have documented a high prevalence of malnutrition among individuals undergoing chemotherapy (9, 10) and PG-SGA has been validated as a prognostic tool in individuals with advanced cancer (11). However, the need for thorough clinical and patient-reported data frequently limits the use of these all-inclusive techniques in retrospective analysis. PNI and NRI, on the other hand, are objective indices that may be used in large retrospective cohorts since they are derived from routinely available laboratory and anthropometric information. As a result, the current study concentrated on PNI and NRI as practical and repeatable measures of nutritional status in this context, while recognizing that they might not fully capture all aspects of malnutrition evaluated by more thorough instruments.

Malnutrition and chemotherapy toxicity have a multifactorial mechanistic relationship. Malnutrition caused by protein-energy malnutrition results in decreased hematopoiesis, dysfunctional immunity, alteration of drug metabolism and diminished clearance of cytotoxic agents by liver and kidneys. Skeletal muscle loss affects the pharmacokinetics of chemotherapy and increases the exposure of each drug to lean body mass, which results in a greater risk of toxicity. The inflammatory pathways, which are manifested by hypoalbuminemia and neurotic indicators of inflammation, further autobolsticate catabolism and impair physiological stability to systemic therapy. They have been constantly mentioned in systematic reviews and meta-analyses, which found malnutrition and sarcopenia to be important modifiable risk factors of chemotoxicity across solid tumors. The recent research has highlighted the need to adopt standardized diagnostic models (1, 12). A study showed that higher chemotherapy toxicity in patients with advanced lung cancer was independently linked with malnutrition determined by the Global Leadership Initiative on Malnutrition (GLIM) criteria, which justifies the clinical prominence of structured malnutrition assessment (13). Even though this research adds to the evidence base, the research is also limited to lung cancer, and it is not possible to directly apply the results to gastroenteric tumors, in which the nutritional challenges are disease-specific and more severe. Umbrella reviews also confirm that malnutrition, irrespective of the tool used, is always associated with worse clinical outcomes, even though the study populations, types of cancer, and endpoints of toxicity show a high level of heterogeneity (14).

Although the importance of nutrition as a predictor of chemotherapy tolerance is gaining momentum, there are still some important gaps. First, the available literature concentrates on a particular tumor type or more aged groups, and has little evaluation on diverse gastroenteric types of cancer. Second, the nutritional assessment instruments vary to make comparisons and clinical translation more difficult. Third, actual health information on retrospective cohorts is quite limited in most areas, especially in low- and middle-income areas, where the initial malnutrition levels can be greater and the supportive care facilities are more limited. Moreover, although interventional research indicates that nutritional support can prevent toxicity, the relationship between nutritional status before treatment and toxicity in clinical practice is not adequately defined. The current study was intended to determine the relationship that existed between pre-treatment malnutrition and chemotherapy-related toxicities in patients who received systemic therapy due to gastroenteric tumors. In particular, the study will identify the prevalence and severity of malnutrition before chemotherapy and examine how it is associated with the occurrence of chemotherapy-related adverse events, their type and severity (15, 16). Furthermore, the research will aim at investigating the predictability of the pre-treatment nutritional status as an independent predictor of chemotherapy intolerance with the consideration of pertinent demographic, clinical, and disease-related variables. Through the objectives, the study aims at delivering clinically relevant information to support routine nutritional assessment and early risk stratification to enhance treatment tolerance and supportive care strategies in patients with gastroenteric cancer.

## 2 Methodology

### 2.1 Study design and setting

A retrospective cohort investigation was carried out to examine the relationship between pre-treatment nutritional status and chemotherapy-induced toxicity in individuals with gastroenteric malignancies. The study was conducted at a tertiary oncology center via retrospective analysis of regularly collected health records from January 2017 to December 2023. This research was approved by the Ethics Committee of the First Affiliated Hospital of Zhengzhou University, approval number: 2025-KY-1562-001.

### 2.2 Study population

Adult individuals (≥18 years) with histologically confirmed advanced/metastatic gastric or colorectal cancer who underwent systemic cytotoxic chemotherapy were considered for inclusion, representing patients with advanced or metastatic disease. The analysis included 360 subjects with colorectal cancer and 360 subjects with gastric cancer. Subjects were required to have complete baseline anthropometric and laboratory information collected prior to the start of treatment. Exclusion criteria involved previous contact with chemotherapy or radiotherapy, existence of active infection or inflammatory illness at the start, prolonged liver or kidney failure, usage of immunosuppressive treatment, and inadequate documentation of chemotherapy-induced negative consequences.

### 2.3 Baseline clinical and nutritional assessment

Baseline demographic and clinical factors involved age, gender, cancer type, and body mass index (BMI). BMI was computed as weight divided by height squared (kg/m^2^), and undernutrition was classified as BMI < 18.5 kg/m^2^ based on World Health Organization criteria. Laboratory measurements such as serum albumin value and total lymphocyte count, were taken within two weeks before the start of the treatment. Serum albumin less than 3.5 g/dL was considered hypoalbuminemia.

Established composite indices were used to further evaluate the overall nutritional status. The Prognostic Nutritional Index (PNI) was computed utilizing the formula: PNI = [10 × serum albumin (g/dL)] + [0.005 × total lymphocyte count (/mm^3^)], with a cut-off value of ≤45 suggesting malnutrition. The Nutritional Risk Index (NRI) was computed as: NRI = (1.519 × serum albumin [g/L]) + (41.7 × current body weight/ideal body weight). Individuals with severe nutritional risk were identified using an NRI score of ≤83. Both immunological competency and protein reserves were taken into consideration when selecting these indices.

### 2.4 Chemotherapy and toxicity evaluation

In accordance with institutional guidelines, patients with advanced or metastatic colorectal and gastric cancer underwent systemic cytotoxic chemotherapy. In standard clinical practice, regimens differed in terms of drug choice, dosage, scheduling, and duration. Chemotherapy was characterized as a standardized exposure at treatment commencement because of the retrospective design. Chemotherapy-derived toxicity was evaluated throughout therapy and classified as hematological or non-hematological. Hematological damage involved anemia, neutropenia, leukopenia, and thrombocytopenia, while non-hematological harm involved gastrointestinal etc. Negative effects were graded employing the Common Terminology Criteria for Adverse Events (CTCAE). Any grade ≥2 hematological or non-hematological harm that occurred during treatment was considered clinically significant toxicity.

### 2.5 Statistical analysis

Nutritional indicators and baseline features were summarized using descriptive statistics. Baseline demographic and nutritional factors were summarized using descriptive statistics. Frequencies and percentages were used to represent categorical variables, and mean ± standard deviation was utilized for displaying continuous variables. Multivariable logistic regression models were constructed individually for gastric cancer and colorectal cancer groups to determine independent predictors of chemotherapy-derived toxicity. Different multivariate analysis was carried out for hematological and non-hematological toxicity. Adjusted odds ratios (ORs) with 95% confidence intervals (CIs) were reported. Variables were incorporated in the multivariable analysis based on clinical significance and previous research relating nutrition to chemotherapy tolerance. A *p*-value of less than 0.05 was considered statistically significant. SPSS software (Version 26) was used for all analyses.

## 3 Results

### 3.1 Study population and baseline characteristics

**Figure 1:**
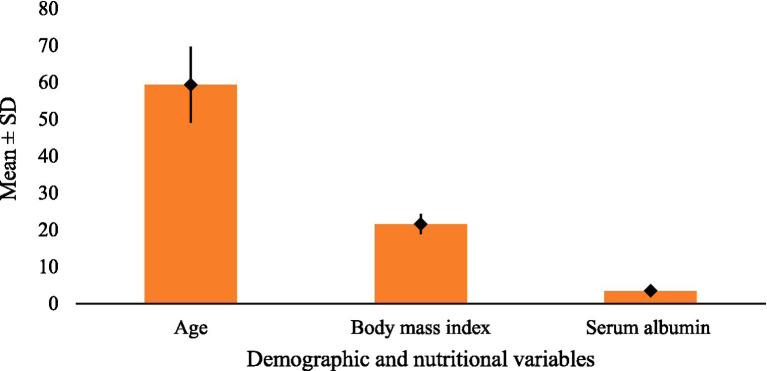
Baseline demographic and nutritional characteristics of gastric cancer patients.

**Figure 2:**
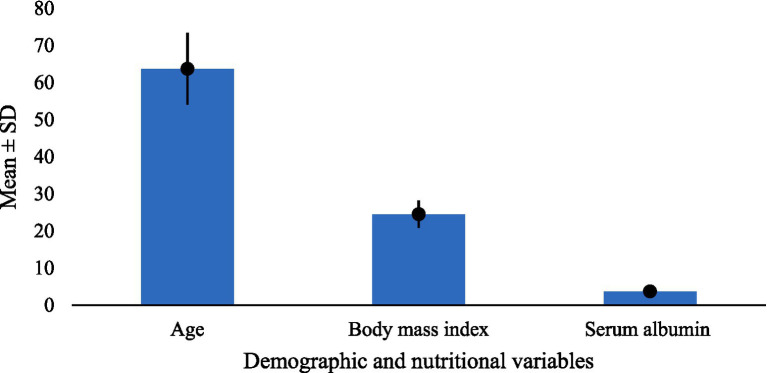
Baseline demographic and nutritional characteristics of colorectal cancer patients.

**Figure 3:**
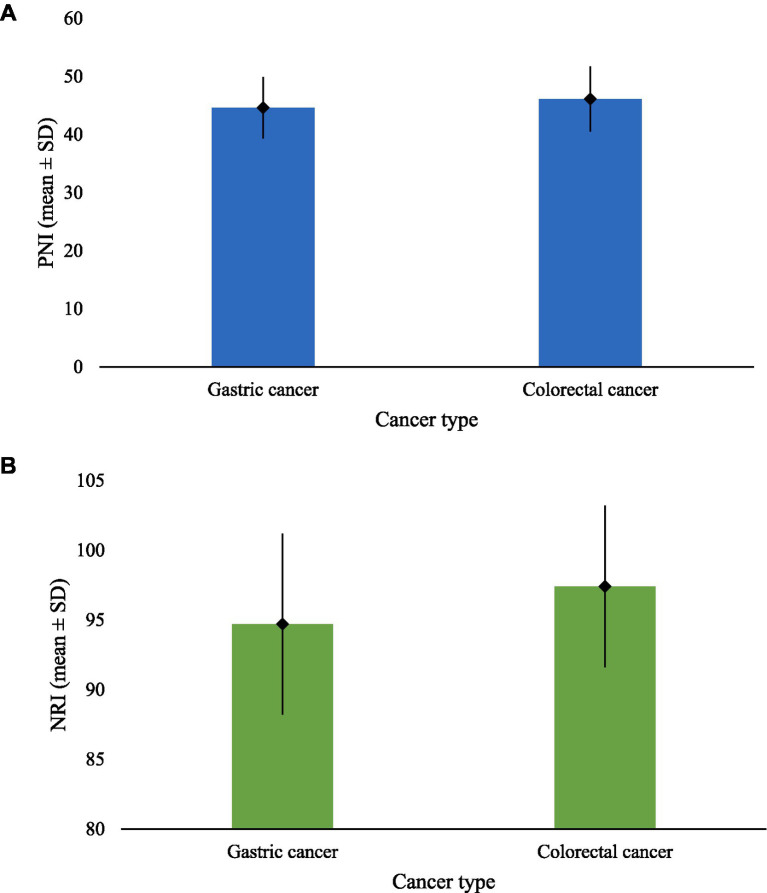
**(A)** Prognostic nutritional index (PNI) by cancer type. **(B)** Nutritional risk index (NRI) by cancer type.

This retrospective cohort investigation involved 720 individuals with advanced or metastatic gastroenteric malignancies receiving systemic chemotherapy, comprising 360 individuals with colorectal cancer and 360 individuals with gastric cancer. Table 1 provides baseline nutritional and demographic data. The study cohort involved a majority of male subjects and covered a wide adult age range. Prior to therapy, a portion of individuals were found to be underweight based on their body mass index values. A significant percentage of individuals were also diagnosed as hypoalbuminemic at baseline. Moreover, a substantial number of the subjects had malnutrition prior to the start of chemotherapy, according to the Prognostic Nutritional Index (PNI) and Nutritional Risk Index (NRI) values (Figures 1, 2, 3).

**Table 1:** Baseline demographic and nutritional features of study participants.

Characteristic	Gastric cancer (n = 360)	Colorectal cancer (n = 360)
Age (years), mean ± SD	59.3 ± 10.3	63.7 ± 9.7
Male sex, *n*(%)	217 (60.3%)	198 (55%)
Body mass index (kg/m^2^), mean ± SD	21.54 ± 2.75	24.5 ± 3.7
Serum albumin (g/dL), mean ± SD	3.43 ± 0.44	3.73 ± 0.45
Prognostic Nutritional Index (PNI), mean ± SD	44.6 ± 5.3	46.1 ± 5.6
Nutritional Risk Index (NRI), mean ± SD	94.7 ± 6.5	97.4 ± 5.8

### 3.2 Chemotherapy-induced toxicity

Chemotherapy-derived toxicity was commonly noted throughout the course of treatment. Table 2 summarizes the identified adverse effects, both hematological and non-hematological. Anemia, neutropenia, leukopenia, and thrombocytopenia were among the anomalies associated with hematological toxicity. Fatigue, mucositis, gastrointestinal complaints, and other negative outcomes related to therapy were included in non-hematological toxicity. During chemotherapy, a significant number of individuals had at least one adverse event. Although non-hematological toxicities were also frequently recorded, a significant portion of reported negative consequences were hematological toxicities. These results show that among the study group, medication-related toxicity was a prevalent clinical problem.

**Table 2:** Chemotherapy-associated toxicity.

Toxicity type	Gastric cancer, *n* (%)	Colorectal cancer, *n* (%)
Hematological toxicity	179 (49.7%)	227 (63.1%)
Non-hematological toxicity	137 (38.1%)	202 (56.1%)

### 3.3 Pre-treatment nutritional status

Pre-treatment nutritional status was assessed employing anthropometric, biochemical, and composite indicators such as BMI, serum albumin values, PNI, and NRI (Tables 3, 4). Before chemotherapy, a portion of patients had underweight status, which is characterized as BMI < 18.5 kg/m^2^. Another prevalent finding was hypoalbuminemia, which denotes lower baseline blood protein levels. A significant percentage of patients had poorer PNI readings, which were indicative of decreased combined nutritional and immunological indices prior to starting chemotherapy. NRI-based classification also determined subjects across a range of nutritional vulnerability, involving patients satisfying requirements for severe nutritional impairment.

**Table 3:** Pre-treatment malnutrition classification (gastric cancer).

Nutritional classification	Category	Gastric cancer, *n* (%)
BMI < 18.5 kg/m^2^	<18.5 kg/m^2^	53 (14.7%)
Hypoalbuminemia	<3.5 g/dL	202 (56.1%)
PNI ≤ 45	≤45	180 (50.0%)
NRI ≤ 83	≤83	10 (2.8%)

**Table 4:** Pre-treatment malnutrition classification (colorectal cancer).

Nutritional classification	Category	Colorectal cancer, *n* (%)
BMI < 18.5 kg/m^2^	<18.5 kg/m^2^	55 (15.3%)
Hypoalbuminemia	<3.5 g/dL	107 (29.7%)
PNI ≤ 45	≤45	156 (43.3%)
NRI ≤ 83	≤83	55 (15.3%)

### 3.4 Associations between baseline variables and chemotherapy toxicity in gastric cancer

**Figure 4:**
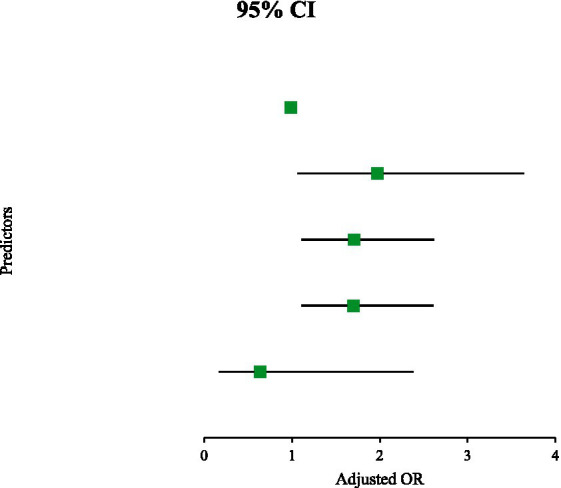
Forest plot of adjusted odds ratio.

Multivariable logistic regression analyses were carried out to assess relationships between baseline features and chemotherapy-related toxicity among individuals with gastric cancer (Table 5). Age at the beginning of treatment was linked to non-hematological toxicity; in adjusted analyses, the risks of negative outcomes increased with age (*p* = 0.001). However, Age and hematological toxicity did not show a statistically important connection (Figure 4).

**Table 5:** Multivariable logistic regression analysis of independent predictors of chemotherapy toxicity (gastric cancer).

Predictor	Toxicity type	Adjusted OR	95% CI (lower)	95% CI (upper)	*p*-value
Age (per year)	Non-hematological	1.03	1.01	1.06	0.001
Age (per year)	Hematological	0.99	0.97	1.01	0.45
BMI < 18.5 kg/m^2^	Non-hematological	0.82	0.44	1.53	0.53
BMI < 18.5 kg/m^2^	Hematological	1.97	1.06	3.64	0.03
Hypoalbuminemia (<3.5 g/dL)	Non-hematological	0.93	0.60	1.44	0.75
Hypoalbuminemia (<3.5 g/dL)	Hematological	1.71	1.11	2.62	0.01
PNI ≤ 45	Non-hematological	0.76	0.49	1.17	0.21
PNI ≤ 45	Hematological	1.70	1.11	2.61	0.01
NRI ≤ 83	Non-hematological	1.11	0.30	4.18	0.87
NRI ≤ 83	Hematological	0.64	0.17	2.38	0.51

Hematological toxicity was linked to being underweight according to BMI parameters. In the adjusted model, patients with a BMI of less than 18.5 kg/m^2^ had increased risks of hematological side effects after chemotherapy (*p* = 0.03). The relationship between BMI and non-hematological toxicity was not statistically significant. Hematological complications were also linked to initial serum albumin levels. An elevated risk of hematological negative effects was associated with hypoalbuminemia at the beginning of therapy. In multivariable model, decreased PNI was also linked to hematological toxicity. However, these links were not seen for non-hematological toxicity. Nutritional Risk Index categorization did not reveal statistically noteworthy relationships with either hematological (*p* = 0.51) or non-hematological toxicity (*p* = 0.87) in adjusted analysis for this group.

### 3.5 Associations between baseline variables and chemotherapy toxicity in colorectal cancer

Multivariable logistic regression analyses were also conducted for subjects with colorectal cancer (Table 6). In adjusted models, age was linked with both hematological and non-hematological toxicity and the likelihood of negative consequences increased with age. Hematological toxicity was linked to underweight condition as determined by BMI criteria. After controlling for other factors, the relation between lower BMI and non-hematological toxicity was not statistically significant. Baseline serum albumin levels were connected with both hematological and non-hematological complications. Individuals with hypoalbuminemia showed increased odds of negative outcomes during chemotherapy in adjusted models (*p* = 0.01). Decreased PNI was linked with hematological toxicity but was not significantly related with non-hematological harm.

**Table 6:** Multivariable logistic regression analysis of independent predictors of chemotherapy toxicity (colorectal cancer).

Predictor	Toxicity type	Adjusted OR	95% CI (lower)	95% CI (upper)	*p*-value
Age (per year)	Non-hematological	1.04	1.01	1.07	0.001
Age (per year)	Hematological	1.03	1.01	1.06	0.001
BMI < 18.5 kg/m^2^	Non-hematological	1.66	0.85	3.25	0.14
BMI < 18.5 kg/m^2^	Hematological	2.00	1.02	3.91	0.04
Hypoalbuminemia (<3.5 g/dL)	Non-hematological	1.90	1.14	3.17	0.01
Hypoalbuminemia (<3.5 g/dL)	Hematological	1.85	1.12	3.04	0.01
PNI ≤ 45	Non-hematological	1.07	0.68	1.68	0.77
PNI ≤ 45	Hematological	1.90	1.21	2.97	0.005
NRI ≤ 83	Non-hematological	2.35	1.19	4.66	0.01
NRI ≤ 83	Hematological	2.50	1.29	4.85	0.007

**Figure 5:**
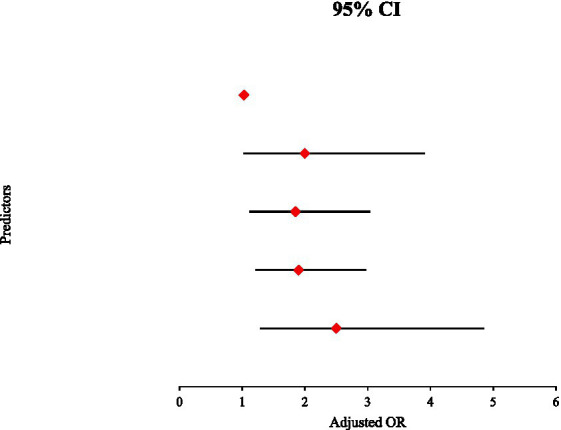
Forest plot of adjusted odds ratio.

In multivariable models, both hematological and non-hematological damage were linked to serious nutritional risk as determined by NRI ≤ 83 (*p* = 0.007). Chemotherapy-associated negative events were more likely to occur among individuals who met this criterion (Figure 5).

In conclusion, patients with gastrointestinal cancers experienced injuries from treatment. Adjusted evaluations revealed highly significant relationships between documented negative consequences and baseline nutritional parameters, including anthropometric, biochemical, and composite indices.

## 4 Discussion

This retrospective cohort study portrays a close relationship between pre-treatment nutritional condition and the experiences of chemotherapy-related toxicity in gastroenteric cancer patients. Based on several nutritional indicator variables, such as: BMI, serum albumin, PNI, and NRI, the results indicate that malnutrition during chemotherapy pre-treatment is prevalent and clinically significant, especially in terms of hematologic toxicity. Such outcomes help to emphasize the significance of systematic nutritional evaluation before starting treatment of gastric and colorectal cancer patients. In the current cohort, almost half of patients with gastric cancer and over 60% of patients with colorectal cancer had been hematologically morbid indicating that treatment related adverse effects continue to be a significant clinical burden. This is consistent with the findings of previous study which showed that frail elderly individuals undergoing chemotherapy are at an increased risk of developing severe toxicity and early mortality especially when nutritional impairment is an issue. Even though the present study was not limited in older adults, age proved to be an independent predictor of toxicity, particularly non-hematological toxicity in gastric cancer, and both types of toxicity in colorectal cancer, which is in line with the idea that physiological susceptibility is augmented by nutritional deficits with age to aggravate treatment tolerance (17). Age-related increases in the likelihood of chemotherapy-related toxicity could be partially explained by older patients decreased physiological reserve. Previous research has demonstrated that elderly people who have poor nutritional status such as low BMI, hypoalbuminemia, sarcopenia, or frailty are more vulnerable to side effects from medication. These results imply that hunger and ageing may work together to reduce chemotherapy tolerance (1, 3, 17, 18).

One of the most important findings of this study is that in both subgroups of cancers, the underweight status is in consistent correlation with the hematological toxicity. The odds ratio of developing hematological complications were almost twice in patients with a BMI less than 18.5 kg/m^2^. This observation is biologically plausible and aligns with existing evidence outlining how loss of lean body mass and sarcopenia affect bone marrow reserve (as impaired), drug metabolism and immune function (as impaired), which make one prone to myelosuppression with chemotherapy (18). Also, another study indicated that sarcopenia occurs in the gastrointestinal cancer patients undergoing chemotherapy and relates to functional deterioration and susceptibility to treatment, which supports the clinical significance of the low BMI and muscle wasting observed in our analysis (19). Hypoalbuminemia at baseline was another good predictor of hematological toxicity in gastric and colorectal cancer patients and also with non-hematological toxicity in colorectal cancer. Not only the nutritional reserves, but also the systemic inflammation and hepatic synthetic capacity is reported in serum albumin. These results are in line with prior evidence demonstrating that inflammation- and nutrition-based indices that use albumin are strong prognostic factors in chemotherapy patients. The observed associations may be explained by the increased exposure to free drugs, altered pharmacokinetics, and decreased protein binding associated with low levels of albumin, which is a mechanistic explanation of the findings (20).

The value of nutritional assessment in predicting health was further enhanced by composite indices. Hematological toxicity, in both types of cancers, was independently related with a low Prognostic Nutritional Index (PNI ≤ 45). PNI combines albumin with lymphocyte count, thus, providing a summary of nutritional and immune conditions. This is in line with the results previous study which stressed that immune-nutritional vulnerability is another factor that leads to chemotherapy intolerance and poor outcomes (17). It is possible that in our study the lack of a significant correlation between PNI and non-hematological toxicity is due to immune-nutritional impairment having a stronger impact on bone marrow suppression compared to other immune-specific toxicities, which is also supported by prior studies. Notably, a strong relationship was observed between severe nutritional risk as characterized by NRI ≤ 83 and chemotherapy toxicity in colorectal cancer as it was linked with hematological and non-hematological adverse events. This result builds on the narrative review which identified that poor nutritional state had a negative impact on chemotherapy adherence and dose intensity in gastrointestinal malignancies. This could be explained by the fact that the higher predictive value of NRI in colorectal cancer patients in the current study could be due to differences in the nutritional reserve of patients at baseline, metabolic requirements, or even treatment of gastric and colorectal cancer (21). Although these results demonstrate the predictive significance of the chosen indices, it is important to keep in mind that PNI and NRI mainly represent biochemical and anthropometric components of nutritional status. Therefore, some clinically significant aspects of malnutrition, such as food intake and functional status, were not assessed in this investigation. Accordingly, a high burden of malnutrition and its correlation with unfavorable clinical outcomes have been shown in earlier research employing structured nutritional assessments in patients undergoing chemotherapy (9–11), which is consistent with the general pattern found in this study.

In addition to toxicity prediction, the systematic review and meta-analysis allows the general clinical implications of nutritional status. They have shown that dietary interventions have the capacity to lower chemotherapy toxicities and improve quality of life indicating that malnutrition is not just a risk factor, but also a factor which can be changed. When combined with our findings, this evidence allows concluding that the implementation of early nutritional screening and intervention as the part and parcel of oncologic care of patients with gastroenteric tumors is justified (22).

Pre-therapy nutritional condition is another variable determining chemotherapy-induced toxicity in gastroenteric cancer patients, especially gastric cancer. The results obtained using anthropometric, biochemical, and composite nutritional indicators demonstrate that malnutrition prior to treatment is widespread and highly related to hematological toxicity, but its correlation with non-hemoglobin toxicity is more selective. One interesting finding in the study is that malnutrition is high before chemotherapy particularly in gastric cancer patients of which over half of them had hypoalbuminemia and half of them had low PNI scores. These results are consistent with the systematic review and meta-analysis, which found that the digestive system cancers are some of the malignancies with the heaviest burden of nutritional risks. The authors highlighted that the location of tumors, general inflammation, and deficient intake are predisposing factors to the early involvement of nutritional degradation, which substantiates the susceptibility presented in the current cohort of gastric cancer (23).

Underweight (BMI < 18.5 kg/m^2^) was found to be an independent predictor of hematological and not non-hematological toxicity in the multivariable analysis. This selective association indicate that low body mass mainly works on the bone marrow endurance as opposed to organ-related toxicities. Other similar mechanistic insights have shown that low muscle mass and impaired nutritional status are strongly associated with the functional decline, and low physiological reserve in patients receiving systemic therapy because of esophagogastric tumors (24). Even though their paper concentrated on quality of life and sarcopenia, the correlation between the inadequate nutritional condition and the decreased treatment tolerance follows the pattern of the hematological susceptibility observed in our cohort. Another good predictor of hematological toxicity in patients with gastric cancer was hypoalbuminemia. Not only nutritional reserves are reflected by serum albumin, but inflammatory and metabolic stress as well. This observation is in line with the previous retrospective cohort study which indicated that reduced PNI, which is primarily due to hypoalbuminemia, was strongly linked with myelosuppression caused by chemotherapy in patients with gastric cancer. Combined, these results support the idea that poor protein condition predisposes the patient to defective hematopoietic fever in the course of cytotoxic therapy (25).

The predictive value of nutritional assessment was further supported in this study by the Prognostic Nutritional Index. Hematological toxicity was significantly correlated with A PNI ≤ 45 but there was no significant correlation between A PNI ≤ 45 and non-hematological toxicity. The selective effect is consistent with prior findings and suggests that the primary effect of immune-nutritional impairment to the form of bone marrow suppression could be rather than gastrointestinal or systemic side effects. Such findings highlight the usefulness of composite measures of nutritional and immunological values combining the use of single anthropometric measures (25). The Nutritional Risk Index, in its turn, failed to provide significant correlation with hematological or non-hematological toxicity in cancer patients of the gastric type. It could be explained by the rather low percentage of patients with the problem of severe malnourishment based on NRI criteria within this group. However, evidence suggest that malnutrition is still high in population-based levels in patients who survived colorectal cancer all over the world, which explains that the effect of NRI and other indexes might be dependent on the stage of the disease, the setting of treatment, and the type of cancer (26).

Non-hematological but not hematological toxicity among gastric cancer patients was independently linked to age. This implies that aging can result in enhanced susceptibility to both functional and organ-specific adverse effects and not necessarily affect the marrow suppression. These results are in line with the general geriatric oncology results that age interacts with nutritional and functional impairment to increase treatment intolerance, especially in non-hematological fields. The wider clinical implications of malnutrition are well captured in the literature besides the toxicity prediction. Malnutrition has been shown to be associated with worse cancer-specific and non-cancer-related mortality in patients with colorectal cancer patients, which reinforces the idea that the state of nutritional impairment has long-term adverse effects on treatment outcomes in excess of short-term treatment toxicity (27). Likewise, the assessment based on the principles of the PG-SGA showed that the malnutrition burden is high in the case of chemotherapy patients and that the clinical significance of the early identification of the nutritional condition in the colorectal cancer patients was huge (28). Notably, there is an emergent body of interventional evidence that the nutritional risk is modifiable. The meta-analysis and systematic review demonstrated that gastrointestinal symptoms and quality of life are reduced with the help of systematic nutritional interventions during cancer treatment (29). The digital health intervention trial also suggested the feasibility of active monitoring of nutrition in complicated gastrointestinal cancer management, which proved the translational relevance of finding high-risk patients before starting chemotherapy (30).

As evidenced in the current research, there is a significant and steady interrelation between the nutritional status at baseline and the probability of chemotherapy-related toxicity among patients with colorectal and gastrointestinal cancer. Our results indicate that increasing age, hypoalbuminemia, low body mass index, and impaired prognostic nutritional index (PNI) and high nutritional risk as established by nutritional risk index (NRI) are the predictors of hematological and non-hematological adverse events in the course of chemotherapy with a clinical meaning. These findings support the increased appreciation of nutrition as one of the primary determinants of treatment tolerance in oncology. Age turned out to be a single predictive of hematological and non-hematological toxicity, and the risk rose gradually with respect to the age. This finding is in line with the available literature that underscores the influence of age on physiological alterations, decreased organ reserve, and altered pharmacokinetics that make older individuals susceptible to chemotherapy intolerance. Aging is often followed by sarcopenia, chronic inflammation, and subclinical malnutrition, which in combination make people less resistant to anticancer treatments. Our results would add to this idea by showing that age is a factor of toxicity risk independent of nutritional indices, indicating a cumulative risk of being older in a patient (31). It was adjusted that low BMI, especially underweight status, had a significant role in the hematological toxicity but not non-hematological toxicity. This is partly consistent with previous findings indicating that weight loss and low body mass as factors leading to more complications associated with chemotherapy especially bone marrow suppression. The non-significance of non-hematological toxicity in adjusted models indicates that that the anthropometric measure alone might not be sufficient to determine the complexity of nutritional deficits affecting non-hematological outcomes, highlighting the shortcomings of anthropometric measures in their own right (32).

One of the strongest predictors of negative outcomes in our case was serum albumin. Both hematological and non-hematological toxicity were found to be strongly linked to hypoalbuminemia even after adjusting the confounders. This is in line with previous findings that albumin is a surrogate of nutritional reserve, systemic inflammation, and severity of the disease. Similar findings have been reported, indicating that malnutrition categorized according to comprehensive assessment tools was closely connected with adverse clinical outcomes in gastrointestinal malignancies. These findings are supported by our results and also serve to underscore the fact that the low concentrations of albumin can be the measure of the poor protein synthesis, the increased inflammatory load, and the diminished ability to withstand cytotoxic stress (33).

The composite nutritional indices were found to add prognostic value to the single parameters. Lower PNI had a significant relationship with hematological toxicity, indicating that the combination of immunological and nutritional malfunction makes a person vulnerable to bone marrow toxicity. This observation aligns with models emphasizing the importance of immune-nutritional interdependence in the treatment response and toxicity in the case of gastrointestinal cancer patients. The lack of substantial correlation between PNI and non-hematological toxicity could be used to indicate that there are different biological processes that occur when such adverse events take place (34). It is crucial to note that severe nutritional risk processed by NRI less than 83 was a significant predictor of both hematological and non-hematological toxicity and one of the most significant predictors in adjusted analyses. This confirms the idea that multidimensional nutritional indices are more suitable to indicate the metabolic and functional conditions of cancer patients on a global level. Small nutritional risk at early stages must be identified in order to carry out the timely nutritional intervention throughout the cancer treatment spectrum of prehabilitation to rehabilitation. Our results support this suggestion empirically and show that patients who satisfy high-risk criteria of NRI are significantly more prone to suffer harm related to chemotherapy (31). Individuals with advanced and metastatic gastroenteric malignancies were assessed as a single cohort since the main objective of the current study was to examine the overall relationship between pre-treatment nutritional status and chemotherapy-related toxicity rather than variations based on disease stage.

### 4.1 Strengths

There are a number of strengths of this study. To begin with, the sample size 720 is relatively big and increases the statistical power of the study and its generalizability to the context of gastroenteric malignancies. The two populations of gastric and colorectal cancer can be used to conduct cancer-specific analyses which provide more precise data on how nutritional status affects chemotherapy toxicity. Second, the research used effective nutritional measurements, combining anthropometric (BMI), biochemical (serum albumin), and composite indices (PNI and NRI), which in combination give a multidimensional perspective of pre-treatment nutritional status. Third, this is due to the standardized approach to toxicity grading that is administered through the Common Terminology Criteria for Adverse Events (CTCAE) in which chemotherapy-induced adverse events are uniformly and clinically relevant assessed. Lastly, performing an individual multivariate logistic regression on hematological and non-hematological toxicities, as well as correcting the results based on clinically significant covariates, enhances the credibility of the determined correlation between malnutrition and treatment-related complications.

### 4.2 Limitations

Despite its benefits, this study has a number of drawbacks. First, the study’s retrospective design makes it vulnerable to recall and reporting bias since clinical exposures and results might not have been regularly or fully recorded in medical records. As a result, the quality and completeness of the available data determine how reliable the results are. Additionally, using pre-existing information makes it more difficult to fully account for confounding variables because crucial covariates could not have been methodically recorded, which could result in residual confounding. The observed relationships may potentially have been impacted by selection bias and inaccurate documentation. Toxicity effects may have been impacted by significant clinical variables that were not completely recorded, such as patient comorbidities and differences in treatment regimens. Because baseline comorbidity data was not uniformly recorded in the retrospective records, the adjusted analyses were unable to fully evaluate this. However, treatment-related side effects, chemotherapy tolerance, and nutritional status may all be impacted by underlying medical issues (e.g., diabetes mellitus). This should be taken into account when analysing the correlations found in this study.

The investigation did not include information on treatment-related parameters, such as the kind of chemotherapy regimen, cumulative exposure, and dose adjustments, which could have impacted the assessment of toxicity outcomes. These factors may serve as surrogate indicators of treatment exposure and regimen severity and are known to affect chemotherapy-associated toxicity. Their absence makes it more difficult to determine whether the reported toxicities were caused entirely by baseline nutritional status or in part by differences in treatment intensity. As a result, care should be taken when interpreting the relationships found in this study, especially when considering how they may affect oncologic efficacy.

Additionally, performance status and symptom-associated variables, including nausea, were not included. These factors may influence both nutritional status and treatment tolerance and are not captured in the nutritional indices used in this investigation (35, 36). The research was carried out in one tertiary care facility, and it could limit the external validity of the results to other healthcare facilities or populations. Moreover, there was no evaluation of the dynamic alterations in nutritional status with chemotherapy and only the baseline nutritional measurements were taken, which could have underestimated the cumulative effect of malnutrition. Despite its usefulness, the NRI may not be as sensitive in identifying intermediate nutritional risk in this population. Furthermore, the lack of thorough nutritional assessment instruments like GLIM or PG-SGA limits the measurement of multifaceted elements of malnutrition. Moreover, separate stage-based analyses comparing locally progressed and metastatic disease were not conducted in this study. Future research may examine possible variations in chemotherapy tolerance and nutritional status based on disease stage. Also, information regarding nutritional support during therapy, like Ryle’s tube feeding, enteral feeding, and parenteral nutrition, not consistently documented in the retrospective data and hence were excluded from the current analysis.

### 4.3 Future recommendations

The need to reduce bias and enhance generalizability should focus on prospective and multicenter research in future studies. Nutritional status longitudinal measurements during chemotherapy may be useful in understanding the time equation between malnutrition and toxicity. Interventional trials of study of preemptive nutritional support, such as dietary counseling or supplementation, could assist in developing causal connections and workable plans to mitigate complications associated with the treatment procedures. Also, the extent of incorporating molecular or inflammatory biomarkers with conventional nutritional indices would potentially increase the predictive values of chemotherapy toxicity. An individualized nutritional intervention could also be enhanced by stratifying patients according to particular chemotherapy regimens and tumor subtypes, which would also improve clinical outcome.

## 5 Conclusion

Baseline nutritional insufficiency was associated with chemotherapy-induced toxicity in individuals with gastric and colorectal malignancies. Objective indicators of malnourishment determined before therapy initiation were constantly linked with higher vulnerability to negative outcomes, suggesting lowered physiological reserve during cytotoxic treatment. The found connections highlight the medical significance of regularly evaluating nutritional status as part of pre-chemotherapy assessment, especially to identify individuals who may otherwise be deemed acceptable for standard regimens but are at increased risk of toxicity. These findings encourage a more proactive incorporation of nutritional evaluation into oncologic decision-making, with possible ramifications for dose choice, monitoring intensity, and supportive care. Future prospective and interventional research is required to ascertain whether early dietary intervention might enhance therapy endurance and reduce toxicity without losing oncologic effectiveness.

## Data Availability

The raw data supporting the conclusions of this article will be made available by the authors, without undue reservation.
